# The Story of the Insane from Year to Year

**Published:** 1893-10-28

**Authors:** 


### THE STORY OF THE INSANE FROM YEAR TO YEAR.

(Sixth Series.)

### IPSWICH BOROUGH ASYLUM.

Here the numbers fell from 279 to 263. There were 63 admissions, 37 recoveries, and 33 deaths. Table III. gives these numbers thrown into percentages, and the recoveries are put down at 63·7 and the deaths at 12·2, but 37 recoveries on 63 admissions would work out at 58·7. In 17 cases hereditary influence caused the insanity, and in 4 drink is responsible for it. Eleven of the deaths were due to brain diseases, 10 to consumption, and 1 to enteritis. Dr. Rowe calls attention "to the fact that light, cheerful, and pretty surroundings very materially assist in the treatment of the insane, and have a distinctly beneficial effect on the conduct and temper of the patients." The average weekly cost per head is 9s. 3d.

### SALOP AND MONTGOMERY ASYLUM.

Here an increase of 30 patients has to be noted during the year 1892. There were 221 admissions—65 recoveries and 85 deaths. Put into percentages in the usual way these numbers yield 29·41 recoveries and 11·1 deaths. Hereditary influence accounts for 24 of the admissions and drink for 26. Forty-four of the deaths were caused by some form of brain disease and consumption for 11, while enteric fever and enteritis account for 1 each. The wages of the attendants and their leave of absence have both been increased. The visitors record "their cordial appreciation of the services of the medical superintendent of the other officers of the institution." The weekly rate of maintenance is 8s. There are 27 private patients, and the charge for these is 15s. a week each.

